# Doing involvement: A qualitative study exploring the ‘work’ of involvement enacted by older people and their carers during transition from hospital to home

**DOI:** 10.1111/hex.13327

**Published:** 2021-10-02

**Authors:** Natasha Hardicre, Jenni Murray, Rosie Shannon, Laura Sheard, Yvonne Birks, Lesley Hughes, Alison Cracknell, Rebecca Lawton

**Affiliations:** ^1^ Yorkshire Quality and Safety Research Group, Bradford Institute for Health Research, Temple Bank House Bradford Royal Infirmary Bradford UK; ^2^ School of Health and Community Studies Leeds Beckett University Leeds UK; ^3^ Social Policy Research Unit University of York York UK; ^4^ Leeds Centre for Older People's Medicine, St James' University Hospital Leeds Teaching Hospitals NHS Trust Leeds UK; ^5^ School of Psychology, Faculty of Medicine and Health University of Leeds Leeds UK; ^6^ Present address: Laura Sheard, Department of Health Sciences University of York York UK

**Keywords:** care transitions, involvement work, older people, patient involvement

## Abstract

**Context:**

Being involved in one's care is prioritised within UK healthcare policy to improve care quality and safety. However, research suggests that many older people struggle with this.

**Design:**

We present focused ethnographic research exploring older peoples' involvement in healthcare from hospital to home.

**Results:**

We propose that being involved in care is a dynamic form of labour, which we call ‘involvement work’ (IW). In hospital, many patients ‘entrust’ IW to others; indeed, when desired, maintaining control, or being actively involved, was challenging. Patient and professionals' expectations, alongside hospital processes, promoted delegation; staff frequently did IW on patients' behalf. Many people wanted to resume IW postdischarge, but struggled because they were out of practice.

**Discussion:**

Preference and capacity for involvement was dynamic, fluctuating over time, according to context and resource accessibility. The challenges of resuming IW were frequently underestimated by patients and care providers, increasing dependence on others post‐discharge and negatively affecting peoples' sense and experience of (in)dependence.

**Conclusions:**

A balance needs to be struck between respecting peoples' desire/capacity for non‐involvement in hospital while recognising that ‘delegating’ IW can be detrimental. Increasing involvement will require patient and staff roles to be reframed, though this must be done acknowledging the limits of patient desire, capability,and resources. Hospital work should be (re)organised to maximise involvement where possible and desired.

**Patient/Public Contribution:**

Our Patient and Public Involvement and Engagement Panel contributed to research design, especially developing interview guides and patient‐facing documentation. Patients were key participants within the study; it is their experiences represented.

## 1 INTRODUCTION

Shorter hospital stays, which support patient preference to be at home and reduce strain on acute care resources, can result in people needing ongoing care, often requiring multi‐agency input.^1^ Unfortunately, it has been estimated that one in 10 patients experiences an adverse event in the immediate post‐discharge period.^2^ Alongside the stress associated with being hospitalised,^3^ harm is also caused by systemic issues, such as inadequate care provision across care boundaries, including across settings.^2^ Williams et al.^2^ (p. e829) suggest that ‘there is great potential for significant reduction in harm from even small improvements in this process (of transition from hospital to home)’.

Patient involvement has been suggested as a way of improving the quality and safety of patient care,^4^, ^5^ particularly by contributing to enhanced system functioning.^6^ This is especially relevant when care delivery and system functioning are challenging, for example, when delivering care across settings, boundaries, and at transitional moments such as when patients return home after a hospital stay.

Following Murray et al.,^7^ we consider involvement to be nuanced, dynamic and relational, changing over time and influenced by context and interaction. In this way, patient involvement has the potential to operate in multiple ways to influence system functioning. For example, Schubert et al.^8^ suggest that by navigating a ‘fragmented system’, patients/caregivers can ‘identify and prevent mistakes from happening, and participate in improving their care’ by enabling care co‐ordination across multiple settings and providers. Likewise, O'Hara and Lawton^9^ argue that patients have the potential to act as ‘information conduits’ across settings, thereby improving safety and reducing harm. However, despite being the highest users of the National Health Service (NHS), research shows that older people, in particular, struggle to be involved in their care,^10^, ^11^, ^12^ therefore minimising potential contributions towards patient safety. Moreover, little is known about the *desired* involvement of older people from their perspective across the transition from hospital to home, especially over time.

Within this study, we explored what older people (aged 75+) understood by ‘involvement’, how they ‘did’ involvement and where there were opportunities for enhanced involvement, during and after a hospital stay, in ways that were acceptable to them.

## 2 METHODS

We undertook a longitudinal focused‐ethnography^13^ exploring the involvement and experience of older patients from hospital admission to 3 months post‐discharge.^14^ This enabled us to explore ‘involvement at transitions’ as a specific phenomenon through inquiry and engagement with older people in everyday life and over time, something limited within the current body of literature. We adopted an inductive, pragmatic approach, with analysis being data‐driven and interpretive. Specifically, we aimed to move past individual accounts of experiences and perceptions to identify ‘underlying ideas, assumptions, and conceptualisations’^15^ from the corpus of data, including from multiple participants, while remaining rooted within individual accounts and experiences.

### 2.1 Research question

Can older people be more involved in their care? If so, how and in what ways?

This was the first study in our programme of work seeking to improve the quality and safety of care through development of an intervention designed to increase patient involvement, specifically in older populations. Consequently, inherent in the programme design, and this study's research question formulation, was a theoretically informed assumption that older people *can* be more involved in their care and that being so will have a positive impact on that care.^4^, ^5^, ^6^, ^7^, ^8^ However, we wanted to remain open to older peoples' experiences and preferences around involvement and so we also aimed to explore:

To what extent do older people feel involved in their care? What are their perspectives on this?

Where are the opportunities for older people to be more involved in their care, should they desire this?

To what extent do older people feel *able* to be (more) involved in their care? What has, or would help them to, feel able to be (more) involved in their care?

We felt that being open to different experiences and perspectives about involvement, while being committed to making improvements, would increase the likelihood of developing a person‐centred intervention, sensitive to the lived experiences and preferences of those we were seeking to help.

#### 2.1.1 Sample and setting

Community‐dwelling adults aged 75+ were the target study group as they are most likely to experience variability in care at transitions. End‐of‐life patients were excluded because they tend to have dedicated post‐discharge care pathways. Likewise, people being discharged to live in residential care were excluded as they were likely to experience different care at transitions to their community‐dwelling peers due to readily accessible postdischarge support.

A total of 32 patients aged 76–99 years were recruited from six hospital wards across multiple specialities from three hospitals within two NHS trusts in Yorkshire, North of England (see Table 1). A total of 18 family members were also recruited. We purposively recruited a diverse group: individuals of different ethnicities (with translation assistance), people with and without relatives performing a ‘carer’ role, and a variety of ages including the ‘oldest old’ (aged 85+). Patients with cognitive or language impairments and those lacking capacity to consent were included, provided they had suitable support.

**Table 1:** Patient demographic details

Mean age	79
Median age	84
Age brackets	
75–79	*N* = 7
80–89	*N* = 15
90–99	*N* = 10
Male	*N* = 14
Female	*N* = 18
Asian: Pakistani	*N* = 2
White: Other White background	*N* = 2
White: English/Welsh/Scottish/Northern Irish/British	*N* = 28

Recruitment and initial interviews/observations and early follow‐ups were completed in hospital, during which staff were also spoken to informally during observation work. Further contacts with patients took place in intermediate care settings and in patients' own homes.

#### 2.1.2 Recruitment

Patients were recruited shortly after admission to the hospital. Initially, decisions about which patients to approach were opportunistic; sampling became more purposive as the study progressed.

Senior ward staff helped identify eligible patients and made initial approaches. Researchers discussed the study with patients and their family, if present; all those approached were given a participant information sheet and the opportunity to ask questions as they considered participation. All participants provided written informed consent and were assigned pseudonyms to maintain their anonymity. The study was approved on 8 March 2017 by Wales 7 NHS research ethics committee (17/WA/0057).

#### 2.1.3 Data collection

Semi‐structured interviews were the primary means of generating data, supplemented by observations,^14^ ‘go‐along interviews’^14^, ^16^, ^17^ and relevant contextual information from patients' care records. We looked at care records after initial interviews to explore the extent to which people knew and understood the reason for their admission. We also looked at care records when people moved to new care facilities and were unable to recall information about transfer dates and next steps. This facilitated accurate data capture and enabled timely follow‐up. All patients consented to this access.

Each contact with patients was recorded as a field visit (FV). One hundred and sixty FVs were conducted in total (by authors N. H., R. S. and L. H). The fewest number of FVs with a participant was three; the highest was nine. The timing of FVs varied according to patients' care journeys, but broadly occurred at admission, before/at discharge, shortly after discharge, several weeks post‐discharge, and 3 months post‐discharge and/or at readmission.

Interviews were audio‐recorded, where possible. Observations were recorded through field notes. Researcher interpretation and key reflections were noted after FVs; these were used to provide contextual information during analysis.

#### 2.1.4 Data analysis

Tacit analyses were done throughout the period of data collection by authors N. H., R. S. and L. H., who each reviewed their own data and met regularly throughout the project to discuss key ideas. Additionally, N. H. listened to voice recordings/read transcripts generated by each researcher. A thematic analysis^15^, ^18^ was led by N. H., with regular input and sense‐checking from R. S. and L. H. to ensure that the identified themes represented the whole data set.

**Figure 1:**
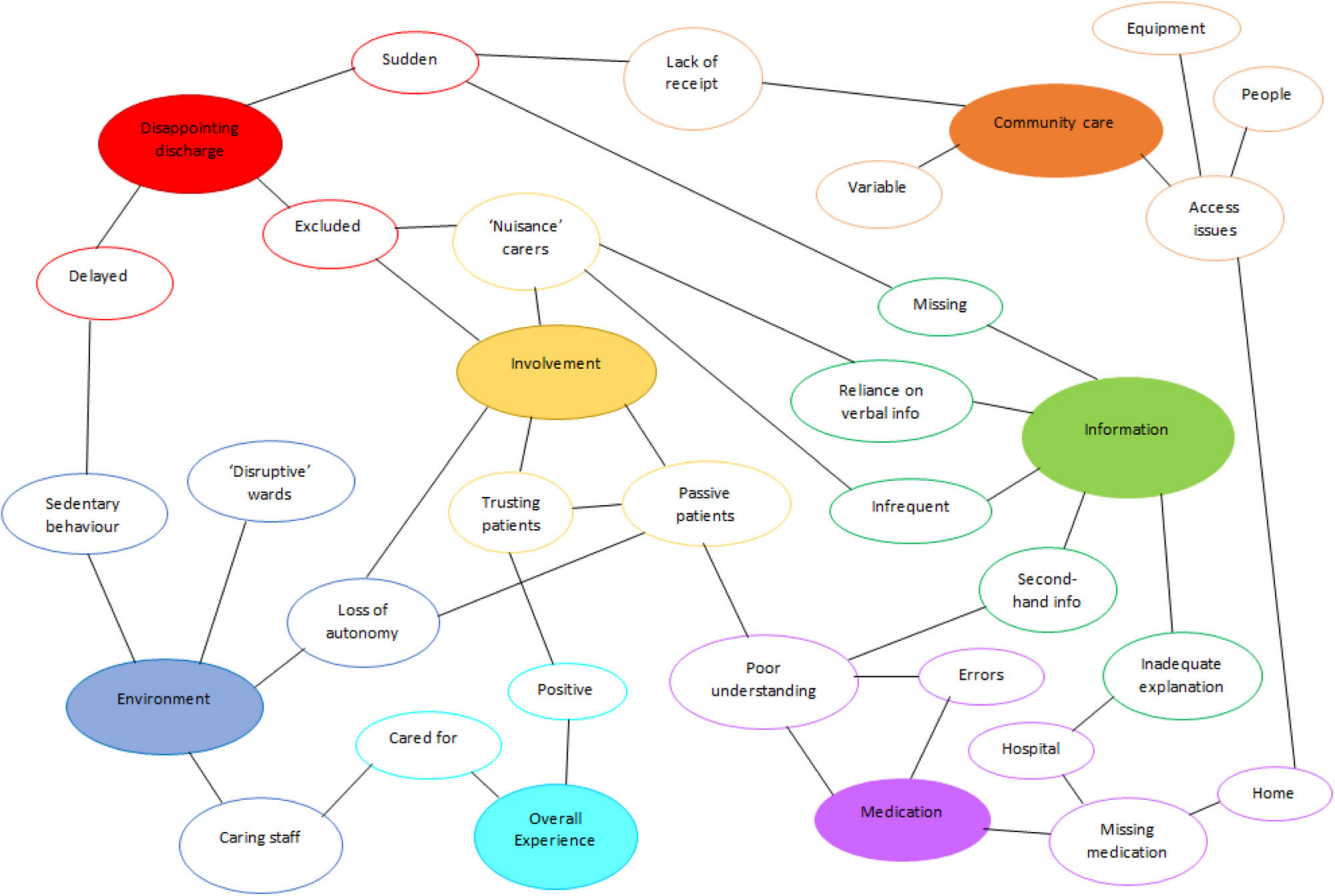
Themes and subthemes

Key ideas were organised by N. H. into categories and subcategories, followed by identifying patterns and relationships between these categories. Similarities and differences between categories were used to construct themes and subthemes and the relationships between them (see Figure 1). Additionally, comparison of themes across FVs for each participant was done to explore continuity and change in perspective and experience over time.

## 3 FINDINGS

### 3.1 Involvement work

For health care professionals (HCPs) to deliver care to patients, work needs to be done. That is, decisions need to be made, activities undertaken, and tasks need to be completed—often by multiple people, over time, and within different contexts. Care delivery work is, predominantly, visible and acknowledged *as* work—HCPs are employed to carry out these tasks, for example. However, our study also found that *receiving* care required patients (and/or their relatives) to do ‘work’ too, including decision‐making, undertaking activities, and completing tasks. We consider the work that people do as, and on behalf of, patients to be the ‘labour’ of involvement, conceptualised in this paper as ‘involvement work’ (IW).

We propose that IW has three dimensions—cognitive, emotional and instrumental (see Table 2). These dimensions often coexisted and were experienced and/or enacted simultaneously. IW also operated along a continuum, with people moving between states of ‘Non‐involvement’ at one end and ‘Involvement’ at the other. Being involved was a dynamic, interactional, and relational process. For example, during her first admission, Pearl (91) wanted HCPs to ‘look after her’—she was tired and felt that being in hospital provided respite from doing everyday tasks that she normally managed (patient‐desired non‐involvement). However, HCPs became concerned that Pearl was unable to manage independently at home because she was both reticent and struggling to mobilise independently on the ward. They recommended Pearl return home with a care package or move into residential care; Pearl was asked to decide between these options (cognitive IW). This triggered feelings about loss of independence and worry about losing her home (emotional IW). Resolved to stay at home, Pearl became as active as possible on the ward (instrumental IW moving her along the continuum towards ‘involvement’), determined to prove to HCPs that she was motivated and capable (emotional IW). In the meantime, three of Pearl's daughters were having ongoing discussions with each other and with HCPs to decide upon and make post‐discharge arrangements (cognitive and instrumental IW).

**Table 2:** Dimensions of involvement work

Cognitive	Emotional	Instrumental
This tended to involve activities such as decision‐making, weighing up options, planning for future care, and understanding information and processes. This type of involvement often included interaction with others, especially healthcare professionals, frequently as providers of information	Emotional involvement work is about the emotions that are generated and ‘managed’ before, after or when receiving care or when enacting other forms/dimensions of involvement work	This included undertaking activities, or co‐ordinating or integrating work, such as chasing up test results, medications or appointments
	Interactions with others was a key aspect of emotional involvement work	It often included interactions with others, especially when navigating systems and co‐ordinating activities, although it also included tasks that could be undertaken independently
Examples		
Ray (76) spent time postdischarge researching a new medication to decide if he was happy to take it. He then discussed his concerns with his GP	Trevor (84) was motivated to be involved in decision‐making about his discharge because he was very anxious to get home to his wife, who lives with dementia and struggles to manage without him.	Philip (81) chased up his missing medication postdischarge by contacting the GP and community pharmacy to arrange a new prescription
Katherine (83) did not want to take her new medication, but she discussed this with her consultant at a postdischarge outpatients' appointment. Together, they agreed that she would take it to manage her health condition	Martin (83) built good relationships with staff during a long hospital stay. This facilitated trust and positive affect between the staff and Martin, giving him confidence and an increased willingness to engage with therapy, despite it being challenging and painful	Shirley (93) realised that hospital ward staff were busy and so she walked to the toilet independently instead of asking for a commode at the bedside

For Pearl, enacting IW was challenging—sometimes impossible—and frequently invisible. For example, on the admitting ward, Pearl began going to the en‐suite toilet independently because she could not find her buzzer, though staff rarely knew about it (invisible IW). However, after moving wards, Pearl no longer knew where the toilet was, and this made it difficult to go to the toilet alone; moreover, staff on this ward encouraged Pearl to use a commode instead of walking to the toilet at the end of the ward (challenging IW). Then, during another ward move, Pearl's walking frame was misplaced, resulting in Pearl being unable to walk around independently at all (impossible IW due to lack of resources). Pearl rarely communicated any of her feelings or difficulties to others because she did not want to be ‘a nuisance’ (hidden IW). Moreover, Pearl's daughters told us how difficult it was to get enough information from HCPs to make necessary decisions and arrangements (cognitive and instrumental IW). Getting information required persistent chasing (instrumental IW) and caused stress and frustration (emotional IW), none of which they communicated to Pearl because they did not want to worry her (invisible IW).

Pearl's case is a good example of how IW undertaken by patients/relatives, and the resources required to enact this, can remain hidden from others. Consequently, unlike the work of HCPs, IW remained largely invisible to and unacknowledged by HCPs, patients, and families alike. The consequences of this were twofold. First, ‘entrusting’ IW was common. We found that because the hospital healthcare system was geared towards the visible and acknowledged work that HCPs do, this set up expectations about the roles and behaviours of healthcare staff and patients operating within the system: Most patients desired minimal involvement during their hospital stay, seeing much of the cognitive and instrumental work as the responsibility of HCPs and/or their families. Hospital processes seemed to tacitly support these expectations by promoting and prioritising care delivery work, and minimizing or failing to support opportunities for involvement.

Second, we found that enacting IW required resources, which was also largely unacknowledged by staff and patients, although it was frequently articulated as an issue by relatives. Because the provision of and access to resources were variable and/or unequally distributed, doing IW was sometimes challenging, even when desired.

### 3.2 Entrusting IW

This involved actively or passively minimizing participation in one or more types of involvement and took two forms: outsourcing and delegating. Both forms were common within our cohort of patient participants. For some people, outsourcing and delegating were enduring experiences; for others, they were transient experiences, usually adopted during episodes of acute ill‐health.

#### 3.2.1 Outsourcing

‘Outsourcing’ involved handing over responsibility for IW to other people, primarily to HCPs. This approach was one of ‘do [task] *for* me’. This was often due to patient expectations about the role of HCPs as knowledgeable decision‐makers, capable care providers, and the people whose job it was to perform tasks in hospital. However, ‘choosing’ to outsource IW to HCPs was often an interplay between patient preference and ‘going along’ with usual hospital processes, which tended to undertake activities on behalf of patients as standard—for example, HCP kept and dispensed medication, even when patients usually managed this. Many patients were willing to accept this because they trusted hospital staff.

I go along with it, it's the hospital, they know what they're doing. (Lillian, 80)

However, sometimes, outsourcing activities became more about ‘compliance’ than a patient's desire to avoid doing the task themselves. This led patients to outsource IW to HCPs because they wanted to be ‘good patients’.

I do as I'm told; I don't want to be a nuisance. The staff have got so much to do. (Jeannie, 89)

#### 3.2.2 Delegating

‘Delegating’ was also a means of handing over responsibility to others. This was often partial and usually to unpaid caregivers, for example, relatives. This approach was one of ‘do [task] *on my behalf*’, and patients typically remained influential while delegating IW. For example, patients frequently informed family members of their preferences and relatives communicated these preferences to care providers; relatives often became proxies for patients. Sometimes family members explicitly acknowledged this role, describing themselves as ‘advocates’ for their relative. For most relatives, however, doing involvement activities on behalf of patients was undertaken as an obligation; their role as ‘delegate’ was treated matter‐of‐factly and often remained unacknowledged by both patients and their family members.

Entrusting IW to others was the most frequent and desired form of involvement in hospital and was supported by standard hospital care processes, within which hospital staff undertook many cognitive and practical aspects of involvement for patients as part of caring for them. However, desire to entrust IW to others was not static and it varied, primarily according to time and location (see Table 3).

**Table 3:** Involvement work across time and location

Time	Admission early hospital stay	Hospital stay predischarge and discharge period	Postdischarge
Location	Hospital	Hospital	Home or intermediate care (IC)
Involvement work	Outsourcing	Variable: Delegating; desire to contribute to decision‐making; outsourcing; desire to resume autonomy with activities of daily living (ADLs)	Variable: Desire to/resuming autonomy; outsourcing to social care; delegating
Summary	Patients often relinquished control of their treatment and care to healthcare professionals (HCPs) at admission. Patients viewed themselves as ‘non‐experts’ and HCPs as experts. This was the case even when patients were used to doing these activities for themselves at home	Some people wanted to resume normal daily activities as they started to feel better, though opportunities were often limited. Others, however, were happy to continue being cared for by staff and continued to outsource responsibility, even when encouraged to start resuming some autonomy	Desired and actual involvement work varied postdischarge. Some patients resumed autonomy with few problems; others struggled to readjust to independent living. Sometimes, this was because they still felt unwell. Others, however, had adapted to institutional living, had become deconditioned, and were unable to manage at home. This was often a surprise to patients and relatives, despite a hospital stay where most ADLs had been managed for them and sedentary behaviour was common.
	Early in admission, outsourcing decisions and care‐related activities were often done because patients were not able, or did not want, to do these for themselves. Some expressed relief that staff were undertaking activities for them, experiencing their hospital stay as respite	As discharge planning continued, many patients became more interested in being involved in decision‐making; being able to decide place of residence was a concern across the sample. Some patients felt able to contribute to discussions themselves. However, many patients preferred to delegate their involvement to relatives.	Sometimes resuming involvement work was made more difficult by inadequate information, especially when prescriptions had changed, and patients were unaware of this. This caused confusion and unintentional noncompliance
			Patients were more likely to continue entrusting care‐related activities to others when they were in IC settings (outsourcing); had packages of care (outsourcing); or family involvement (delegating). Regardless of setting, almost all participants were happy to continue to outsource medical decisions, especially those who had good relationships with their GP
Participant examples/quotes	*‘*That's a medical decision, isn't it? I have nothing to do with it… I don't know zilch’, (Leslie, 84; acute medical unit for older people [AMUfOP])	Pearl (91) did not speak during care planning meetings with her social worker. Instead, Pearl outlined her wishes to her daughters for them to discuss on her behalf. Pearl felt that her daughters were more effective in these meetings than she could be—this was due to hearing loss and a lack of confidence in her own ability to navigate a complex system.	Leslie (84) did little for himself in hospital, but once back at home he recommenced cooking for himself, managing his medicines, and tracking and chasing up appointments
	‘I know the kids are worried because I'm in hospital… but I'm having a bit of a rest’. (Pearl, 91; AMUfOP)		‘[We thought] it would all fall into place once she got here [home], but that's not the case, she's refusing to walk, she's fallen twice so far because she can't get in or out of her bed, she's struggling. She was ringing for a cup of tea’. (Lillian's niece ‐ Lillian, 80)
			*‘*I mean, I don't want a miracle, I just want them [the doctors] to explain it to me and then I can sort things, you know. Because I mean now, before, how many tablets? Now I'm taking about six I think, I was taking three before then’. (Katherine, 83)

### 3.3 Maintaining IW

Not all patients wanted to outsource IW during their hospital stay. However, those wishing to retain autonomy frequently had to *resist* hospital processes; this often required undertaking additional work.

#### 3.3.1 Resisting processes

Although there was some variation between wards, standard processes for managing and caring for patients were broadly similar across locations and allowed minimal room for individual patient preferences. Ward‐based environments were homogeneous, with limited scope for personalisation; for example, patients had almost no input on ward temperature, lighting, care schedules, and choice about food and drink provision was limited. This was a source of frustration for some patients, especially as they began to feel better or during lengthier hospital stays.

I said to a nurse this morning, ‘Could I please go down … and get my own water? I've managed to cope with that [laughs]. But can I? No! No, she wouldn't let me make tea, but then she wouldn't bring me any tea! [Laughs]’. (Katherine, 83; Stroke rehabilitation ward)

Katherine became so frustrated by everything being done for her that she started handwashing her clothes, crockery, and cutlery in the sink in her room, despite staff telling her this was unnecessary. For Katherine, resuming activity was crucial to her self‐identity as an independent person.

Another process common across wards was ‘falls prevention’. Most patients were considered by HCPs to be at moderate‐high risk of falling and reported being encouraged to remain in bed or seated at the bedside. After speaking to HCPs about this, a senior nurse said that while, ideally, they would enable patients to remain as active as possible, this was extremely resource intensive and they rarely had sufficient staffing levels to facilitate and support physical activity. Consequently, minimizing physical activity felt like the safest option for patients at risk of falling. Many participants were compliant, not wanting to be bothersome, or were worried about falling themselves. However, some patients actively chose to ignore instructions, for example, choosing to walk independently, even when encouraged or told they should sit down. For example, Ray (76) declined to use the wheelchair brought to him when he moved around the ward. HCPs were persistent in offering the wheelchair, despite Ray's confidence and ability in walking independently, and what was initially a ‘decline’ had to become a more active ‘refusal’.

### 3.4 Resources for IW

Involvement was often resource intensive, frequently requiring knowledge and information, social support, and material resources (see Table 4). Some patients had limited access to resources such as informal support, and this resulted in an increased reliance on health and social care services; interestingly, patients relying on formal care provision often struggled to resume IW post‐discharge.

**Table 4:** Resources for involvement work

Information and knowledge	Support	Material resources
Most people we spoke to wanted more information. For some patients, being informed was a key means of being involved; not being informed provoked anxiety or frustration. Some people needed information to make decisions or plans for future care, especially carers.	Support networks were a key resource for many patients. Sometimes. support networks provided additional support to enable a patient to readjust to living independently postdischarge. For example, Shirley outsourced her IW while in hospital but was keen to regain autonomy at home. However, being subject to disruptive hospital routines and feeling unwell at the point of discharge meant that readjusting was challenging. However, Shirley mobilised her support networks to help her in the immediate postdischarge period until she could fully resume her normal activities.	Involvement work (IW) was sometimes financially costly. Relatives of patients reported spending a lot of money on hospital parking costs to visit relatives. However, attending hospital was necessary for gaining information and being involved in decision‐making and future care planning. Relatives often felt that these costs were unavoidable if they wanted to be involved.
People who wanted to maintain their IW were more likely to seek information than people who outsourced their care to others:		
‘I always ask, I'm a great believer in asking, asking questions, and they may not know the answers but they'll get to know the answers for you, you know? So I find that it's like, life's less complicated that way’. (Diana, 78)		Information about postdischarge care was often limited and most patients had few means of accessing additional or correct information when it was missing or inadequate. Access (or not) to resources, such as a computer or internet access, was sometimes instrumental in being able to resolve issues. For example, Doris (99) received a letter asking her to call a telephone number to book a clinic appointment. Unfortunately, the number provided was no longer in service and Doris had no means of contacting the clinic to book an appointment. Compare this with Ray (76), who, when faced with a similar situation, was able to source the correct clinic number using an internet search engine. He not only called to make his appointment but also alerted staff to the error on the letter, who assured him they would change the incorrect information.
Likewise, ‘delegates’ were also likely to be active information‐seekers:	Other patients relied on ongoing family support to stay at home and avoid residential care. Martin's nephew provided help with washing and dressing every morning and evening, enabling Martin (83) to stay at home and reducing burden on Martin's wife, who was unable to provide this type of support because of her own health issues.	
‘You know what I'm like, I interrupt them, I ask questions, I've got to know the inside out of what's it, and, you know, I cause a lot of problems for a lot of people [health care professionals] because I'm just interested, well I need to know the information’. (Serena's daughter—Serena, 92)		
Patients receptive to information, but not active in seeking it out, were less likely to receive information because most staff expected people who wanted information to request it:	Access to social support networks were also necessary when patients wanted to delegate IW, or when tasks needed doing that the patient was unable to do. For example, during his stay in an intermediate care setting, Peter (84) spent 3 weeks in the same pair of hospital pyjamas he was discharged in. Care home staff frequently documented that ‘family need to bring clean pyjamas in’; however, Peter had no family or friends and consequently, no one to provide him with additional clothes.	
*‘*We [nurses] wait until a patient asks [for updates], but don't tend to worry about it at all if they're confused, because they won't take it in’. (Staff nurse, AMUfOP—nonverbatim quote paraphrased from field notes)		
Patients and carers with existing knowledge were often advantaged. Pearl's daughter Tracey, for example, worked as a healthcare assistant in the hospital and was familiar with many hospital processes. Likewise, Philip was a retired pharmacist, which enabled him to spot and avoid a potentially serious medication error during his hospital stay.		

At times, resources needed to be externally provided (e.g., information about medication or expected post‐discharge care, and equipment) and it was problematic when these resources were not supplied. Conversely, active resource provision by HCPs appeared to improve peoples' experiences and facilitated them in managing their health/care post‐discharge. This included resources such as patient‐friendly written information about medications, which enabled people to check what they should be doing once at home or provided the basis for conversations with post‐discharge care providers.

Alongside resources provided by others, the capacities and capabilities of patients themselves could be resources to doing or resuming IW. In particular, feeling confident communicating with HCPs seemed to be a key facilitator for active involvement for both patients and relatives, especially when seeking information, challenging/resisting processes that minimised involvement opportunities, and influencing decision‐making. Overall, we found that patients with access to multiple resources, that is, both their availability and the means to utilise them, were often more effective at maintaining or resuming IW according to their preferences and were more likely to have a positive influence on their care when they did choose to be involved.

### 3.5 Consequences of entrusting or enacting IW

Although maintaining IW was challenging during hospitalisation, it appeared to enable people to resume involvement more effectively post‐discharge than those who entrusted IW to others throughout their stay. Outsourcing, in particular, seemed to contribute to loss of confidence and deconditioning in undertaking activities. This sometimes meant that patients felt less able to manage at home than they had before going into hospital, especially initially. For example, one participant, Mary, attempted to resume her pre‐hospital activities, but had become deconditioned during her hospital stay, resulting in a subsequent fall at home and readmission to hospital. Conversely, we found that participants who maintained active involvement reported ‘getting back to normal’ sooner than people who had outsourced and delegated.

Alongside maintaining capabilities, involvement also influenced subjective experiences of (in)dependence. Crucially, it appeared that congruence between desired and actual involvement was more important to perceptions of (in)dependence than levels of actual dependence. Mary, for example, was happy to outsource tasks and be reliant on others, but was desperate to maintain choice about where she lived; her sense of independence came through being involved in decision‐making about place of residence. It was this that constituted meaningful involvement for Mary. Conversely, Katherine retained autonomy regarding decision‐making and was influential in decisions about discharge planning. However, Katherine felt dependent on others because she wanted to be more involved in her practical care than she could be. Her subjective experience of independence was low because her actual levels of involvement did not match her desired levels.

Lack of resource provision could also impact on post‐discharge experiences by minimising opportunity for involvement in hospital, with visible consequences for patients and staff—missing mobility aids increased reliance on staff to help patients mobilise, for example. However, some problems did not become apparent until patients left hospital and as such were likely to remain invisible to hospital staff, who may not be aware of issues unless problems were significant enough to trigger readmission. For example, having no understanding of new medication is not necessarily a problem while HCPs are dispensing it in hospital. However, once patients become responsible for this post‐discharge, lack of understanding can lead to unintentional medication noncompliance.

Alongside consequences for patients, enacting or entrusting IW had an impact on both care providers and families. For HCPs, active involvement could save them time and reduce care delivery work. For example, patients going to the toilet independently meant that staff time was not needed to help patients. However, some people—especially relatives—felt that they were treated as a ‘nuisance’ by staff when they enacted IW or when they sought out resources for IW. Relatives, in particular, said that because information provision was minimal, they frequently had to seek this out. However, doing so often meant interrupting HCPs during tasks and some people said that staff tacitly communicated displeasure at such interruptions. Information flow was largely controlled and dictated by HCPs and people frequently struggled with this, feeling limited power to effect change or have an influence unless this was facilitated by HCPs.

However, the power dynamics of IW should not be seen as unidirectional. By having the decision on whether to enact or entrust IW to others, patients were able to impact both positively and negatively on the care delivery work that HCPs did and the IW that relatives undertook. The power to say ‘no’ to doing IW was particularly potent; HCPs wanted to (and were also duty‐bound) to care for patients, and family members often felt obliged to provide time, energy, and any financial cost it took to undertake IW on their behalf. IW, then, is always an interplay between people and is often negotiated relationally and interactionally.

## 4 DISCUSSION

The findings of the study suggest that most participants were not actively involved in their care in hospital. While non‐involvement was largely desired during this time, it was also tacitly promoted by hospital processes, which automatically assumed responsibility for most tasks people normally engaged in. ‘Non‐involvement’, then, was often a kind of ‘collaborative accomplishment’ between HCPs and patients: from the point of admission, many patients wanted and expected to ‘outsource’ and ‘delegate’ their IW to others, while care delivered in hospital often failed to enable active patient involvement—even when desired—by doing ‘IW’ on behalf of patients. At times, this seemed to benefit both patients and care providers—patients wanted to be ‘looked after’ and staff wanted to care for patients in ways consistent with hospital processes, which implicitly supported non‐involvement. At times, shortages of care resources, especially staff, also lent itself to patients being uninvolved; some patients sensed that the most helpful role they could play was as a passive patient. Non‐involvement, then, was sometimes a type of ‘collusion’ between patients and HCPs. This may provide some short‐term ‘benefits’, but as noted, it can also result in longer term consequences, especially post‐discharge. These were infrequently anticipated by care providers or patients.

Importantly, involvement preferences were dynamic, varying according to time and context, with some people expressing or demonstrating a desire to resume IW at a later point in their journey, especially post‐discharge. Moreover, the point of discharge marks the moment when patients become responsible for their IW again, because outsourcing cognitive and instrumental IW to HCPs is no longer possible outside a hospital setting. This happens whether resuming IW is desired or not. However, resuming IW was sometimes challenging, especially when people had outsourced/delegated to others, and were therefore out of practice; others lacked resources to be involved in their care. Consequently, opportunities for increasing involvement within this cohort may be difficult without adjusting patient expectations, implementing broader system changes, and ensuring adequate access to resources. A culture of non‐involvement can impact the patient's transition of care in ways often unanticipated by both HCPs and patients. Enhancing involvement may be challenging in hospital, but the consequences could be far‐reaching by enhancing experience and safety post‐discharge.

Increasing patient involvement is likely to require a shift in both expectations (of and about patients and HCPs) *and* in the organisation of the work that goes on in hospitals. For example, patients who demonstrate some capability to do things while in hospital, but are resistant to doing them (preferring to outsource), can be encouraged, reassured, and motivated by staff to consider looking ahead to prepare for their forthcoming independence post‐discharge. Likewise, where staff recognise a patient's desire for autonomy in preparation for resuming life at home, they support rather than resist this. Other potential opportunities include altering hospital processes to facilitate greater involvement where possible, for example, increased patient involvement in medicines management,^19^, ^20^ and engagement with campaigns such as #EndPJparalysis.^21^ Doing so may have ‘knock‐forward benefits’^22^ for both patients—who can gain and maintain skills and confidence—and healthcare services, which could undertake fewer tasks for patients. In agreement with Carman, however, interventions need to be designed to address the factors that impact on patient involvement, including going beyond patient factors such as knowledge or motivation.^23^, ^24^

It is also important to recognise that ‘good’ patient involvement will not look the same for all people and that for some, non‐involvement (e.g., outsourcing) or delegated involvement may be the preferred approach. In these cases, facilitating greater involvement of relatives, where possible, could be beneficial. For other people, ‘passive’ forms of involvement, such as an understanding and acceptance of care and treatment plans, may be ‘adequate’.^22^ This means that information‐sharing between HCPs and patients and their families is vital—not just as a means to active involvement, for example, shared decision‐making, but as the means of involvement itself. Importantly, HCPs may need to take the lead with regard to providing information, as many older people are ‘information receptive,’ but not active in seeking information.^7^ Active information provision may also be required if patients are to be ‘information conduits’ between parts of the system across the transitional journey.^4^

Patient expectations have been identified as a key factor in determining participation, with patient *desire* (to participate) proposed as a prerequisite of participation.^25^ We too found that when patients expect and desire others to do IW on their behalf, they are less likely to participate in their care. This, combined with services geared towards passive patients, creates a ‘perfect storm’ of non‐involvement, much of which may be desired by patients and tacitly welcomed by service providers. Carman argues that organisational characteristics, policies, and practices can (positively) influence patient participation.^23^ However, despite potential system benefits when patients engage in IW, services appear to be predisposed towards non‐involvement. This is often a consequence of the way work is organised, rather than deliberate exclusion by HCPs.

Alongside ‘desired non‐involvement’,^7^ approaches towards more active types of involvement and ability to undertake IW appear to be mediated through access to, and *ability to leverage*, multiple resources and are therefore subject to unequal distribution. Importantly, these include peoples' capacities and capabilities. The concept of ‘patient activation’ has been used to describe the ‘knowledge, skills and confidence a person has in managing their own health and healthcare’; higher levels of activation are promoted as a means of improving health‐related outcomes.^26^ Greene and Hibbard^27^ go as far as to say that ‘patients *should* be more active and effective managers of their health and health care’. In some ways, our study supports such a proposal. We found that the patients in this study most effective at exerting influence and enacting IW in ways meaningful to them were proactive; had existing relevant knowledge; were confident talking to HCPs; and/or were able to resist, challenge, or work around problematic organisational processes within multiple settings. However, we also found that few participants felt able to do these things, especially when unwell. Consequently, it may be useful to consider concepts such as ‘activation’ as a *resource*—which people may or may not have access or ability to leverage at a given time—rather than an attribute of an individual. In agreement with Sinding et al.,^28^ it is important to acknowledge the potential barriers that people can face when attempting to be involved. Otherwise, uncritical promotion of increased patient involvement may serve to aggravate existing health and social inequalities.

Moreover, an individual's (high) level of activation may not be sufficient to enable them to actually undertake more active forms of IW. Shim,^29^ for example, proposes that individuals approach HCPs with ‘a repertoire of cultural skills, verbal and non‐verbal competencies, attitudes and behaviours, and interactional styles' that she refers to as an individual's cultural health capital (CHC). Shim suggests that the CHC individuals bring into consultations and interactions is crucial to how HCPs respond to attempts that people make to be involved in their care and can account for dynamics of unequal treatment between patients, regardless of how capable or competent people may actually be. This is because some cultural resources are more highly valued by clinicians than others, putting those without these resources at a disadvantage.^30^ Likewise, Entwistle and Watt^22^ propose that clinicians who view their patients as ‘capable and trustworthy’ are more likely to facilitate patient involvement and joint working, especially in decision‐making. Sinding et al.^28^ similarly propose that ‘involved patienthood’ requires HCPs to recognise and acknowledge the skills that patients have. This is not only dependent on whether patients have those skills but also on how well patients are able to demonstrate and communicate them to HCPs *and* how willing HCPs are to recognise and acknowledge them. Thus, patient involvement is not only something determined by individual patients but is instead something that is mediated—positively or negatively—through interaction, especially with service providers as individual HCPs, and/or through organisational practices.^7^

Importantly, being ‘involved’ is not only related to achieving particular ends, for example, deciding upon a course of treatment, managing medications, being active and mobile; it can also be crucial to a person's sense and experience of (in)dependence. Secker et al.^31^ suggest that independence is two‐dimensional, encompassing aspects of reliance on others and ‘experienced independence’, which is the self‐assessed perception that a person's degree of choice, social usefulness and autonomy are consistent with that which they desire. Within Secker et al's.^31^ model, a person may be reliant on others and simultaneously experience a high or low sense of independence about their identity and degree of self‐determination. Likewise, someone may have low reliance on others, but experience a high or low level of self‐assessed independence depending on the degree to which they feel they have choice and autonomy. Our findings are consistent with this. In this respect, then, how someone experiences independence is likely to influence what type of IW is meaningful to them. Likewise, the IW people can engage in—especially within institutional settings—is likely to be particularly influential on someone's subjective experience of independence, regardless of how reliant they are on others to do things for them.

For some people, then, lack of involvement and reliance on others will be detrimental to independence, while for others, reliance on others will be consistent with being independent *if* they can retain autonomy over the things that are important to them. Therefore, ‘meaningful’ involvement is not ‘one‐size‐fits‐all’; rather, it requires a person‐centred approach that takes account of a person's desires, ‘psychological make‐up, biography, social context and cultural heritage’,^31^ alongside the resources they have access to.

## 5 LIMITATIONS

As with all research, our study has limitations. We recognise that observational methods and data generated within shared environments may introduce bias into findings. For example, people may change their behaviour while being observed. However, by spending extended periods of time on wards and with participants, we feel that people became comfortable around the researchers, enabling us to capture naturally‐occurring behaviour. We also believe that data generated by observational methods provide insights that go beyond verbal accounts and therefore have utility despite these limitations.^32^ We are also aware that hospital wards offer limited privacy for personal conversations and for patients to express their care experiences, thereby introducing the potential that accounts are limited. However, we took every effort to use private rooms where possible and build trust and relationships with participants, which we believe encouraged honesty. Also, by following up with people post‐discharge, we also provided opportunities for them to share experiences in private spaces, outside of care environments.

We acknowledge that qualitative research is rarely representative, and our findings are therefore not generalisable to all older people transitioning from hospital to home. However, we believe that the in‐depth nature of the work, comprising multiple data generation methods, provides findings that are credible, dependable, and contribute to research in this area. Moreover, our project patient and public involvement groups have repeatedly reviewed the findings at various stages of data collection and analysis. They felt that we captured important themes and perspectives, many of which mirrored their own experiences of being community‐dwelling older adults, all of whom had experienced transitions of care from hospital to home, suggesting transferability of our findings.

## 6 CONCLUSION

Receiving and being involved in care often require patients and families to engage in ‘work’ that remains largely hidden and unacknowledged. Multiple factors influence the involvement that people desire and enact, including patient characteristics, relational dynamics, resource availability, interactions with others, and system processes. However, in hospital, many people ‘entrust’ IW to others and struggle to resume activities. This can result in increased reliance on people and services post‐discharge, alongside a diminished sense of independence. Enhancing involvement could contribute to positive patient experience and safety. Doing so will require encouraging IW when people are reticent, facilitating IW when people show willingness and desire, and resourcing IW to ensure that burden is minimized and inequalities are not aggravated.

## CONFLICT OF INTERESTS

The authors declare that there are no conflicts of interest.

## AUTHOR CONTRIBUTIONS

All authors declare that they made substantial contributions to (a) the conception and design, or analysis and interpretation of data; (b) the drafting of the article or revising it critically for important intellectual content; and (c) approval of the version to be published.

## Data Availability

The data that support the findings of this study are available from the corresponding author upon reasonable request.
